# Multifidelity deep learning modeling of spatiotemporal lung mechanics

**DOI:** 10.3389/fphys.2025.1661418

**Published:** 2025-09-24

**Authors:** José Barahona Yáñez, Daniel E. Hurtado

**Affiliations:** ^1^ Department of Structural and Geotechnical Engineering, School of Engineering, Pontificia Universidad Católica de Chile, Santiago, Chile; ^2^ Institute for Biological and Medical Engineering, Schools of Engineering, Medicine and Biological Sciences, Pontificia Universidad Católica de Chile, Santiago, Chile; ^3^ Department of Aerospace Engineering and Engineering Mechanics, The University of Texas at Austin, Austin, TX, United States; ^4^ Institute for Medical Engineering and Science, Massachusetts Institute of Technology, Cambridge, MA, United States

**Keywords:** lung poromechanics, multi-fidelity neural networks, reduced order modeling, dimensionality reduction, mechanical ventilation, lung mechanics

## Abstract

**Introduction:**

Digital twins of the respiratory system have shown promise in predicting the patient-specific response of lungs connected to mechanical ventilation. However, modeling the spatiotemporal response of the lung tissue through high-fidelity numerical simulations involves computing times that largely exceed those required in clinical applications. In this work, we present a multi-fidelity deep learning surrogate model to efficiently and accurately predict the poromechanical fields that arise in lungs connected to mechanical ventilation.

**Methods:**

We generate training datasets with two fidelity levels from non-linear finite-element simulations on coarse (low-fidelity) and fine (high-fidelity) discretizations of the lungs domain. Further, we reduce the output spatiotemporal dimensionality using singular value decomposition, capturing over 99% of the variance in both displacement and alveolar pressure fields with only a few principal components. Based on this procedure, we learn both the input-output mappings and fidelity correlations by training a reduced-order multi-fidelity neural network model (rMFNN) that leverages the abundant low-fidelity data to enhance predictions from scarce high-fidelity simulations.

**Results:**

Compared to a reduced-order single-fidelity neural network (rSFNN) surrogate, the rMFNN achieves superior predictive accuracy in predicting spatiotemporal displacement and alveolar pressure fields (R^2^ ≥ 93% (rMFNN) vs R^2^ ≥ 75% (rSFNN)). In addition, we show that rMFNN outperforms rSFNN in terms of accuracy for the same level of training cost. Further, the rMFNN model provides inference times of less than a minute, offering speed-ups up to 462× when compared to finite-element numerical simulations.

**Discussion:**

These results demonstrate the potential of the rMFNN lung model to enable patient-specific predictions in acceptable computing times that can be used to personalize mechanical ventilation therapy in critical patients.

## 1 Introduction

Mechanical ventilation (MV) is the standard-of-care therapy for patients suffering from acute respiratory distress syndrome, as it ensures adequate gas exchange in critical conditions (Agrawal et al., 2021). MV played a vital role during the recent COVID-19 pandemic, which affected over 700 million people globally (Dong et al., 2020), with many hospitalized individuals requiring ventilatory support in intensive care units (ICUs) (Petrilli et al., 2020; Grasselli et al., 2020). Despite its massive use, determining optimal, patient-specific ventilator settings remains a major challenge in clinical practice. Suboptimal configurations can lead to adverse outcomes, such as ventilator-induced lung injury, which can significantly worsen the prognosis of the patient (Madahar and Beitler, 2020).

A growing trend in medical translational research is the construction of digital twins, i.e., computational models of the human respiratory system to support the design of personalized ventilation therapies (Zhou et al., 2021; Sun et al., 2024). Finite element (FE) poromechanical models of the lungs, constructed from patient-specific medical images, have recently demonstrated the ability to reproduce the dynamic interplay between lung tissue and airflow during MV. These models can accurately simulate the spatiotemporal mechanical behavior of lung tissue and predict respiratory mechanics within clinically observed ranges (Avilés-Rojas and Hurtado, 2022; Hurtado et al., 2023). Despite their promise, the substantial computational demands of such high-fidelity simulations hinder their practical adoption in time-sensitive clinical settings. Therefore, a key challenge is to accelerate lung model predictions without compromising their accuracy.

A common approach to accelerating computational predictions of complex models is the creation of surrogate models, i.e., computationally-efficient models capable of approximating quantities of interest of high-fidelity simulations. Recent advances in machine learning (ML) have enabled the creation of increasingly powerful surrogate models or emulators in applications ranging from predicting material behavior (Wei et al., 2019) to weather forecasting (Chen et al., 2023) and fluid dynamics (Brunton et al., 2020). However, the effectiveness of ML-based surrogate models heavily relies on the availability of large, high-quality datasets for training and validation—a condition that is often unmet in biomedical and biomechanical applications, where data are typically limited, heterogeneous, and challenging to acquire. Furthermore, for complex physical models, especially those with high dimensionality and computational cost, assembling such datasets can be prohibitively expensive.

To address the previous limitation, multi-fidelity (MF) surrogate modeling has emerged as a promising strategy. By combining the scarce and expensive high-fidelity simulations with a vast number of lower quality but fast to compute simulations, MF models can exploit correlations between these fidelity levels to efficiently approximate the high-fidelity response of complex systems (Fernández-Godino, 2016). In the biomechanical field, Gaussian Processes ( GP s) models have pioneered in MF surrogate modeling, with applications in drug response modeling (Sahli-Costabal et al., 2019), tissue growth dynamics (Lee et al., 2020; Han et al., 2022), arrhythmia prediction (Gander et al., 2022), and global respiratory mechanics estimation (Barahona et al., 2024). However, traditional GP models are generally limited to low-dimensional output predictions and do not scale well for high-dimensional problems (Liu et al., 2018; Gilboa et al., 2013).

Recent deep learning-based (DL) surrogate models via neural networks ( NN s) offer a more scalable alternative for learning the solution fields of high-dimensional problems. Multi-fidelity neural networks (MFNNs) have recently been proposed to approximate complex, high-dimensional outputs arising from finite element models (Aydin et al., 2019; Meng and Karniadakis, 2020; Meng et al., 2021; Guo et al., 2022; Pawar et al., 2022a). Nevertheless, emulating physical systems with millions of degrees of freedom—especially when accounting for multiphysics or time-dependent behavior—remains computationally demanding. This has led to the adoption of dimensionality reduction techniques, which compress large-scale simulation data into compact representations. In physics problems, these methods have been successfully applied in surrogate modeling across spatial (Lee et al., 2018; Eivazi et al., 2020; Conti et al., 2024; Tong et al., 2024), temporal (Bellamine and Elkamel, 2008; Liu et al., 2021), and spatiotemporal domains (Greve and van de Weg, 2022; Fresca and Manzoni, 2022; Schneider et al., 2024). Recent efforts have combined dimensionality reduction with MFNNs to model wind turbine wake flows (Pawar et al., 2022b) and monitor structural health (Torzoni et al., 2023). Despite these advances, the use of multi-fidelity, reduced-order deep learning techniques in modeling complex physiological systems with translational applications in medicine remains underexplored.

In this work, we develop a framework that leverages a state-of-the-art- multi-fidelity neural network with a dimensionality reduction technique to efficiently train and predict the spatiotemporal poromechanical response of human lungs under MV. We evaluate the model performance in predicting displacement and alveolar pressure fields throughout the lung domain. In Section 2, we revisit a continuum poromechanical formulation of the lungs suitable for patient-specific simulation and generate FE simulations at two fidelity levels using fine and coarse mesh discretizations. We then apply a dimensionality reduction technique to transform the high-dimensional spatiotemporal responses into low-dimensional representations via principal components. Based on this, we train a reduced-order multi-fidelity neural network (rMFNN) using both high- and low-fidelity data collected across a range of physiological and mechanical parameters. In Section 3, we assess the accuracy of the dimensionality reduction and the predictive capabilities of the proposed multi-fidelity model, and compare its performance with that of a single-fidelity neural network model. Finally, in Section 4, we discuss the benefits and limitations of the proposed framework and outline directions for future research.

## 2 Materials and methods

### 2.1 Lung poromechanical modeling

To represent the mechanical interaction between airflow and tissue deformation, we follow a continuum poromechanical formulation for continuum lung dynamic simulations (Avilés-Rojas and Hurtado, 2022). This framework considers the lung parenchyma as a continuum deformable porous medium subject to displacement, traction, flux, and airway pressure boundary conditions. Let $\Omega_{0}\in R^{3}$ be the lung domain in the reference configuration, and $\Omega_{t}\in R^{3}$ its current configuration at the time instant t∈R , which is uniquely determined by the deformation mapping $\varphi :\Omega_{0}\times R\to R^{3}$ such that $\Omega_{t}=\varphi \left(\Omega_{0},t\right)$ . The deformation gradient is given by F≔∇φ(X,t) and the Jacobian is defined as J≔det(F) . We assume that alveolar gas and tissue colocate in the lung domain and that their interaction is governed by conservation laws. Neglecting inertial terms and viscous stresses, and assuming incompressibility of both gas and tissue phases, one can show that the material formulation of mass and linear momentum balance read.

$$\text{Div}\left(P\right)+B=0,\;\text{in }\Omega_{0}\times R,$$ (1)

$$\frac{\partial \varphi }{\partial t}+\text{Div}\left(Q\right)=0,\;\text{in }\Omega_{0}\times R,$$ (2)

respectively, where in Equation 1, P is the first Piola-Kirchhoff stress tensor, and in Equation 2, Q corresponds to the material airflow field. Further, we assume that airflow follows Darcy’s law,

$$Q=\frac{1}{\eta }JF^{-1}\kappa F^{-T}\left[-grad\left(P_{\mathrm{alv}}\right)+\rho_{a}F^{T}B\right],$$ (3)

where $P_{\mathrm{alv}}$ in Equation 3 is the material alveolar pressure field, κ=κI is the intrinsic permeability tensor for an isotropic medium with permeability κ , and η represents the gas dynamic viscosity. The term B represents the material body (gravity) force density field. Although gravity is known to influence regional lung mechanics (e.g., dependent vs non-dependent regions), we neglected body forces in this work to simplify the formulation and isolate the effects of ventilator settings on the poromechanical response.

To represent the mechanical behavior of lung parenchyma, we considered a Blatz-Ko type hyperelastic model with strain energy function (Birzle et al., 2019) given by

$$W\left(C\right)=c\left(I_{1}\,\left(C\right)-3\right)+\frac{c}{\beta }\,\left(I_{3}\,{\left(C\right)}^{-\beta }-1\right)+c_{1}\,{\left(I_{1}\left(C\right)I_{3}{\left(C\right)}^{-1/3}-3\right)}^{d_{1}}+c_{3}\,{\left(I_{3}{\left(C\right)}^{1/3}-1\right)}^{d_{3}},$$ (4)

where in Equation 4 C is the right Cauchy-Green tensor, and $I_{1}\left(C\right)$ , $I_{3}\left(C\right)$ are the corresponding invariants of C . The parameters c and β are related to the Young’s modulus E and Poisson’s ratio ν by E=4c(1+ν) and ν=β/(1+2β) , respectively.

### 2.2 Finite element modeling of high-fidelity and low-fidelity lung poromechanics

**FIGURE 1:**
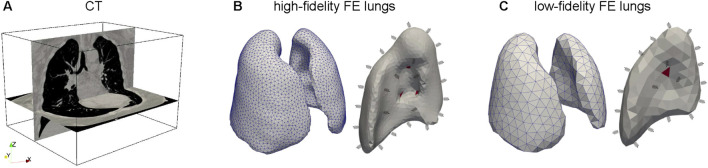
Construction of high-fidelity and low-fidelity lung finite element models. **(A)** From a patient-specific chest computed tomography image, we determine the lung domain, from which we generate finite element tetrahedral meshes for **(B)** the high-fidelity model (fine mesh), and for the **(C)** low-fidelity model (coarse mesh). Red element surfaces denote the regions where boundary conditions are prescribed. The remaining boundary is subject to linear springs to represent the stiffness of the chest wall that surrounds the lung.

Using the described poromechanical formulation, we constructed low-fidelity and high-fidelity finite element models of human lungs under mechanical ventilation. The anatomical domain was extracted from 3D computed tomography (CT) images of human subjects at end-of-expiration, previously reported by our group (Hurtado et al., 2017), see Figure 1A. To create anatomical tetrahedral models, we performed image segmentation and mesh generation following the procedures detailed in (Hurtado et al., 2016). The high-fidelity model resulted in left and right lungs with 45,288 and 59,355 elements, respectively, see Figure 1B). In the case of the low-fidelity model, the left and right lung meshes comprised 2,499 and 3,282 elements, respectively, see Figure 1C). We partitioned each model surface into two boundaries: the airway inlet surface and the visceral pleural surface, whose union comprises the entire lung surface. Based on this boundary partition, we simulated a pressure-controlled ventilation (PCV) mode by prescribing a pressure $\bar{P}$ at the airway inlet boundary, denoted $\Gamma_{aw}$ . We highlight that for the high-fidelity model, we prescribed the pressure in three disconnected airway boundary surfaces, which we determined by considering the surface encompassing bifurcations from the mediastinal surface down to the lobar bronchi (Figure 1B). For the low-fidelity model, we prescribed the airway pressure only on one surface, due to the proximity of airways in the coarser mesh discretization (Figure 1C). To model the interaction of the lung with the chest wall, we considered spring elements with stiffness coefficient $K_{\text{s}}$ to apply a Robin condition of the form $\bar{T}\left(X\right)=K_{\text{s}}\left\{\varphi \left(X\right)-X\right\}$ (Figures 1B,C).

We represented the PCV mode with a time-dependent pressure function $\bar{P}\left(t\right)$ on the airway boundary that resembles the ventilator square wave pressure signals employed in clinical applications. At the onset of inspiration, this function linearly increased until reaching and maintaining a peak inspiratory pressure (PIP), such that $\bar{P}=\text{PIP}$ . Then, the pressure returned to zero during the expiratory phase $\left(\bar{P}=0\right)$ , after which the respiratory cycle repeats. We considered 2 respiratory cycles. To simulate a normal lung at rest, each respiratory cycle considered 1 s of inspiration followed by 2 s of expiration, which is equivalent to a respiratory rate of 20 breaths per minute (Bellani, 2022).

Table 1 shows the baseline values for all of the lung model parameters, which have been shown to deliver a mechanical and global physiological response that is in the range of those reported for normal human lungs (Avilés-Rojas and Hurtado, 2022; Barahona et al., 2024). These parameters encompass the PIP pressure value $\left({\bar{P}}_{\mathrm{PIP}}\right)$ set on the mechanical ventilator, the tissue constitutive model, the porous medium permeability, and the chest wall boundary condition. We also considered a ± 50% range around these baseline values to define a parameter space from which sampling points are randomly drawn during the dataset generation for training the surrogate. As shown in (Barahona et al., 2024), the lung mechanical response is not sensitive to variations in parameters $d_{1}$ and $d_{3}$ , and thus we kept these parameters fixed to avoid redundancy. For simplicity, we define the parameter vector $\xi ={\left[{\bar{P}}_{\mathrm{PIP}},c,\beta ,c_{1},c_{3},k,K_{s}\right]}^{⊤}$ , which will take values on the parameter space $\Xi \in R^{7}$ defined by the cartesian product of the intervals defined in Table 1.

**TABLE 1:** Lung model parameters, baseline values, and intervals for the parameter space considered in lung simulations.

Parameter	Units	Baseline value	± 50% range
${\bar{P}}_{\mathrm{PIP}}$	$cm\;H_{2}0$	6	[3,9]
c	kPa	0.3567	[0.1784,0.5351]
β	−	1.075	[0.5375,1.6125]
$c_{1}$	kPa	0.2782	[0.1391,0.4173]
$c_{3}$	kPa	$5.766\cdot 10^{3}$	$\left[2.8830,8.6490\right]\cdot 10^{3}$
$d_{1}$	−	3	fixed parameter
$d_{3}$	−	6	fixed parameter
k	$mm^{2}/kPa\cdot s$	$1\cdot 10^{4}$	$\left[0.5,1.5\right]\cdot 10^{4}$
$K_{s}$	kPa/mm	$80\cdot 10^{-3}$	$\left[40,120\right]\cdot 10^{-3}$

**FIGURE 2:**
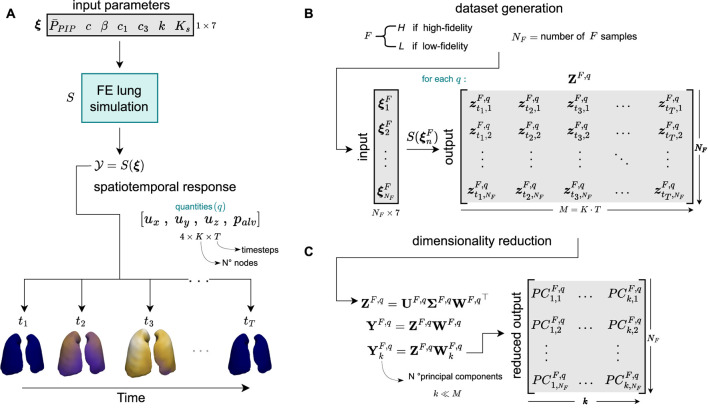
Dataset generation to train the multi-fidelity surrogate model. **(A)** We refer to FE lung simulation as S , which takes as input the parameter vector ξ and produces a spatiotemporal response of the lungs (displacement and alveolar pressure fields) during the respiratory cycle, i.e., z=S(ξ) . **(B)** For each fidelity level F , we perform $N_{F}$ simulations to generate an output dataset matrix $Z^{F}$ , with $N_{H}\ll N_{L}$ . **(C)** We then perform an SVD to reduce the high dimensionality of the spatiotemporal response in both datasets. This process yields a scores matrix $Y_{k}^{F}\in R^{N_{F}\times M}$ of k principal components. We utilize this reduced dataset to efficiently train a multi-fidelity neural network.

For the spatiotemporal discretization of the poromechanics formulation, we employed a backward Euler time-integration scheme and a standard Galerkin multi-field FE discretization (Avilés-Rojas and Hurtado, 2022; Hurtado and Zavala, 2021). This numerical scheme was implemented using the FEniCS library (Alnæs et al., 2015), running all simulations in Python 3.8. We denote the FE simulator as S(ξ) , where ξ is the input parameter vector (Figure 2A). Once a high- or low-fidelity FE lung simulation is completed, we obtain the spatiotemporal response Y=S(ξ) . This response consists of a time series of 4 quantities: the displacement field components $u_{x},u_{y},u_{z}$ and the alveolar pressure $p_{\mathrm{alv}}$ for each node of the mesh. Therefore, for each lung and fidelity level, the response is a 3-D array with shape $Y\in R^{4\times K\times T}$ , where K is the total number of mesh nodes and T is the number of simulated time steps. Each simulation covered 2 respiratory cycles encompassing T=120 time steps. Once the FE simulations are carried out, we can further compute the lung tidal volume, flow, and airway pressure signals as.

$$V_{\text{sim}}\left(t\right)≔\int_{\Omega_{0}}J\left(t\right)d\Omega_{0}-V_{\text{lung},0},$$ (5)

$${\dot{V}}_{\text{sim}}\left(t\right)=\frac{{\partial V}_{\text{sim}}\left(t\right)}{\partial t},$$ (6)

$$P_{\text{aw},sim}\left(t\right)≔\bar{P}\left(t\right),$$ (7)

where in Equation 5 $V_{\text{lung},0}$ is the lung volume in the reference configuration (end-of-expiration) and in Equation 6 $V_{\text{sim}}\left(t\right)$ is the lung volume at time t . Using these signals, we estimate lung mechanics parameters such as the respiratory-system compliance ${\text{C}}_{\text{rs}}$ and airway resistance R from least-squares regression. We refer the interested reader to (Avilés-Rojas and Hurtado, 2022) for further details of the parameter estimation procedure.

### 2.3 Dimensionality reduction of spatiotemporal datasets

We reduce the dimensionality of the simulation datasets via singular value decomposition (SVD). Let F∈{H,L} denote the fidelity label, where H and L correspond to a high or low-fidelity simulation, respectively. To construct the datasets, we sampled $N_{F}$ parameter vectors ${\left\{\xi_{n}^{F}\right\}}_{n=1,\ldots ,N_{F}}$ from the parameter domain Ξ (Figure 2B). For each parameter vector $\xi_{n}^{F}$ we run a lung simulation $\left(S\left(\xi_{n}^{F}\right)\right)$ , obtaining the corresponding spatiotemporal response $Y_{n}^{F}$ . We denote by q any of the four simulated quantities in $Y_{n}^{F}\;\left(u_{x},u_{y},u_{z},p_{\mathrm{alv}}\right)$ . For each $q\in R^{K\times T}$ we apply a flatten operation to produce a horizontally concatenated vector $z_{n}^{F,q}=\left\{z_{t_{1},n}^{F,q},z_{t_{2},n}^{F,q},z_{t_{3},n}^{F,q},\ldots ,z_{t_{T},n}^{F,q}\right\}$ , where $z_{t_{i},n}^{F,q}\in R^{K}$ contains the nodal solutions of q at time instant $t_{i}$ . We note that $z_{n}^{F}\in R^{M}$ , with M=K⋅T . By doing a vertical stack of all the concatenated vectors from the $N_{F}$ simulations, ${\left\{z_{n}^{F}\right\}}_{n=1,\ldots ,N_{F}}$ , we obtain for q an output matrix $Z^{F,q}\in R^{N_{F}\times M}$

$$Z^{F,q}=\left[\begin{array}{c} z_{1}^{F,q}\\ z_{2}^{F,q}\\ \vdots \\ z_{N_{F}}^{F,q} \end{array}\right]=\left[\begin{array}{ccccc} z_{t_{1},1}^{F,q} & z_{t_{2},1}^{F,q} & z_{t_{3},1}^{F,q} & \ldots & z_{t_{T},1}^{F,q}\\ z_{t_{1},2}^{F,q} & z_{t_{2},2}^{F,q} & z_{t_{3},2}^{F,q} & \ldots & z_{t_{T},2}^{F,q}\\ \vdots & \vdots & \vdots & \ddots & \vdots \\ z_{t_{1},N_{F}}^{F,q} & z_{t_{2},N_{F}}^{F} & z_{t_{3},N_{F}}^{F,q} & \ldots & z_{t_{T},N_{F}}^{F,q} \end{array}\right].$$ (8)

From Equation 8, we note that, since four separate output matrices are generated for each lung and each fidelity level, this leads to a total of 4×2×2=16 distinct datasets. We also emphasize that each sampled parameter vector $\xi_{n}^{F}$ implies running a simulation for both the left and right lungs. The high dimensionality of M makes it unfeasible to train a neural network to directly predict the output array $Z^{F,q}$ . Nevertheless, we assume that the simulated lung response should be highly correlated. Spatially, the displacement and alveolar pressure fields in lung tissue are expected to exhibit smooth and coherent variations across adjacent/neighbor nodes $X_{i},X_{j}$ . Temporally, the values of the simulated fields in a certain nodal location $X_{i}$ between consecutive time steps $t_{n},t_{n+1}$ should also exhibit a high correlation. These observations motivate us to apply a dimensionality reduction technique in order to efficiently train the surrogate model. Performing an SVD on each dataset matrix $Z^{F,q}$ (Figure 2C) results in

$$Z^{F,q}=U^{F,q}\Sigma^{F,q}{W^{F,q}}^{⊤},$$ (9)

where in Equation 9, $U^{F,q}\in R^{N_{F}\times N_{F}}$ is an orthogonal matrix whose columns are called the left singular vectors of $Z^{F,q}$ , $\Sigma^{F,q}\in R^{N_{F}\times M}$ is a rectangular diagonal matrix whose values are the singular values of $Z^{F,q}$ , and $W^{F,q}\in R^{M\times M}$ is an orthogonal matrix whose columns are the right singular vectors (principal directions) of $Z^{F,q}$ , with ⊤ as the transpose operator. It can be shown that a scores matrix $Y^{F,q}\in R^{N_{F}\times M}$ of the SVD can be written as

$$Y^{F,q}=Z^{F,q}W^{F,q},$$ (10)

$$=U^{F,q}\Sigma^{F,q},$$ (11)

where in Equations 10, 11, the columns of $Y^{F,q}$ are the scores or principal components. This matrix represents the original high-dimensional data transformed into the new low-dimensional space defined by the principal components. We can obtain a truncated score matrix $Y_{k}^{F,q}\in R^{N_{F}\times k}$ by considering the first k principal components that capture the majority of the variability (upon a certain threshold) of the original spatiotemporal data, with k≪M

$$Y_{k}^{F,q}=U_{k}^{F,q}\Sigma_{k}^{F,q},$$ (12)

$$=Z^{F,q}W_{k}^{F,q}.$$ (13)

We note that Equations 12, 13 represent a reduced or comprised form of the dataset $Z^{F,q}$ . Consequently, each row $y_{n}^{F,q}$ of $Y_{k}^{F,q}$ contains the k principal components (PCs) for the n -th sample, i.e., $y_{n}^{F,q}=\left[PC_{1,n}^{F,q},\ldots ,PC_{k,n}^{F,q}\right]$ Given the reduced response $Y_{k}^{F,q}$ , we can expect to obtain an accurate reconstruction $Z^{F,q}\approx Z_{R}^{F,q}$ by applying the inverse transform in Equation 14:

$$Z_{R}^{F,q}=Y_{k}^{F,q}{W^{F,q}}^{⊤}$$ (14)

### 2.4 Construction of multi-fidelity neural network surrogate models

**FIGURE 3:**
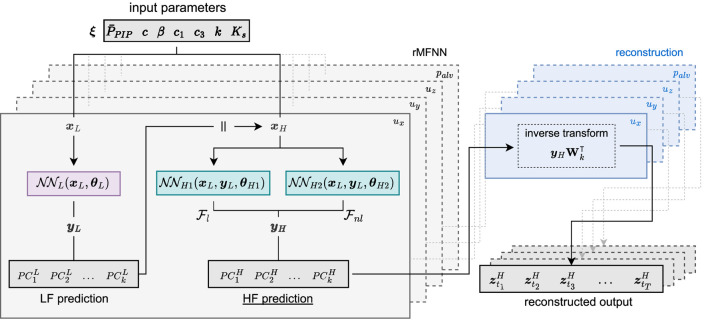
Architecture of the reduced-order Multi-Fidelity Neural Network (rMFNN) surrogate model. The model combines three neural networks to learn the correlation structures between the reduced low- and high-fidelity data: a LF NN trained with abundant low-fidelity data, followed by two HF NNs to capture linear and nonlinear correlations between the reduced-order responses $y_{L}$ and $y_{H}$ . The inputs for $NN_{H1}$ and $NN_{H2}$ is a concatenation (‖) of input parameters $x_{H}$ and the LF output $y_{L}$ . Given a new input, the rMFNN predicts the HF principal components $y_{H}$ , which are then mapped back (reconstructed) to the original space $\left(\left[z_{t_{1}}^{H},z_{t_{2}}^{H},z_{t_{3}}^{H},\ldots ,z_{t_{T}}^{H}\right]\right)$ by applying the inverse SVD transform. Dashed boxes and lines indicate that separate rMFNNs are constructed and trained for each output quantity ( $u_{x}$ , $u_{y}$ , $u_{z}$ , and $p_{\mathrm{alv}}$ ) and for each lung independently.

The primary goal of this work is to develop a DL surrogate model that can take advantage of multi-fidelity data to quickly emulate and predict the spatiotemporal response of patient-specific FE lungs under MV. Specifically, we aim to predict the temporal evolution of displacement and alveolar pressure fields given a specific configuration of the ventilator setting, constitutive model parameters, tissue permeability, and chest wall stiffness (Figure 3). We assume that due to their much lower computational cost, we have considerably more observations from low-fidelity simulations than their high-fidelity counterpart, resulting in $N_{H}\ll N_{L}$ . The multi-fidelity approach builds on the observation that the abundant low-fidelity data, while computationally inexpensive, capture the dominant parametric trends of the system response but introduce systematic errors due to coarse spatial discretization. High-fidelity simulations, while limited in numbers, provide accurate reference solutions of the spatiotemporal response and help correct the biases present in low-fidelity data. Furthermore, we expect that the principal components of the reduced lung responses from high- and low-fidelity datasets ( $Y_{k}^{H,q}$ and $Y_{k}^{L,q}$ ) present a certain correlation to be determined by the multi-fidelity neural network. For simplicity, in this section, we denote the parameter vector ξ as x , thus $\xi^{F}=x_{F}$ . We also refer to a single observation of $Y_{k}^{H,q}$ and $Y_{k}^{L,q}$ simply as $y_{H}$ and $y_{L}$ , respectively. Therefore, we assume that we have high- and low-fidelity datasets of the form $D_{H}=\left\{{\left(x_{H_{i}},y_{H_{i}}\right)}_{i=1}^{N_{H}}\right\}=\left\{X_{H},Y_{k}^{H}\right\}$ and $D_{L}=\left\{{\left(x_{L_{i}},y_{L_{i}}\right)}_{i=1}^{N_{L}}\right\}=\left\{X_{L},Y_{k}^{L}\right\}$ , respectively. For clarity, from now on, we will refer to low-fidelity as LF and high-fidelity as HF.

In the following, we adopt a multi-fidelity architecture specifically developed for physics-based problems Meng and Karniadakis (2020). To find and leverage the relation between low- and high-fidelity data, we consider a generalized autoregressive scheme (Perdikaris et al., 2017) between the LF $\left(y_{L}\right)$ and the HF $\left(y_{H}\right)$ principal components described by

$$y_{H}=F\left(y_{L}\right)+\delta \left(x\right).$$ (15)

In Equation 15, F represents a linear/non-linear mapping from $y_{L}$ to $y_{H}$ , and δ(x) is an additive correction surrogate. Furthermore, the equation can be written as

$$y_{H}=F\left(x,y_{L}\right),$$ (16)

from which we decompose F in

$$F=F_{l}+F_{nl},$$ (17)

where $F_{l}$ and $F_{nl}$ are the linear and nonlinear components, respectively. Therefore, Equations 16, 17 can be written as

$$y_{H}=F_{l}\left(x,y_{L}\right)+F_{nl}\left(x,y_{L}\right),$$ (18)

which combines both linear and nonlinear mappings in the final prediction of HF data. To emulate Equation 18, we employ a composite architecture of three neural networks: a LF NN to learn the low-fidelity data, followed by two HF NNs to capture both linear and nonlinear correlations between low- and high-fidelity data (Meng and Karniadakis, 2020). We summarize this framework as follows.

• The first neural network, $NN_{L}\left(x_{L},\theta_{L}\right)$ , is trained using abundant LF data to capture broad trends and features, acting as a baseline learner in our multi-fidelity setup. Thus, we obtain an approximation for the LF response of the form $y_{L}\approx NN_{L}\left(x_{L},\theta_{L}\right)$ .

• The second neural network, $NN_{H1}\left(x_{H},y_{L},\theta_{H1}\right)$ does not include an activation function in order to learn a linear mapping from the outputs of $NN_{L}$ to the HF targets, thus $F_{l}=NN_{H1}$ .

• The third neural network, $NN_{H2}\left(x_{H},y_{L},\theta_{H2}\right)$ is designed to identify and model any nonlinear relationships between the LF and HF outputs, thus $F_{nl}=NN_{H2}$ .

In this architecture, $\theta_{L}$ , $\theta_{H1}$ , and $\theta_{H2}$ are the unknown parameters (weights and biases) to be learned by each network. Figure 3 shows a diagram with the proposed reduced-order Multi-Fidelity Neural Network (rMFNN). We note that the input for $NN_{H1}$ and $NN_{H2}$ is a concatenation of their corresponding input parameters $x_{H}$ and the LF output $y_{L}$ . Furthermore, we highlight that the approximate HF response of the neural network is given by $y_{H}\approx NN_{H1}+NN_{H2}$ . To learn the network parameters, we optimize the following loss function (Equations 19–21), which aims to minimize the errors across all fidelity levels:

$$L=MSE_{L}+MSE_{H}+\lambda_{L}\Sigma \theta_{L}^{2}+\lambda_{H1}\Sigma \theta_{H1}^{2}+\lambda_{H2}\Sigma \theta_{H2}^{2},$$ (19)

where in Equation 19 we define

$$MSE_{L}=\frac{1}{N_{L}}\overset{N_{L}}{\underset{i=1}{\sum }}{\left({\hat{y}}_{L}-y_{L}\right)}^{2},$$ (20)

$$MSE_{H}=\frac{1}{N_{H}}\overset{N_{H}}{\underset{i=1}{\sum }}{\left({\hat{y}}_{H}-y_{H}\right)}^{2},$$ (21)

i.e., mean squared errors (MSE) from which ${\hat{y}}_{L}$ and ${\hat{y}}_{H}$ are the $NN_{L}$ and $NN_{H}$ predictions, and $\lambda_{L}$ , $\lambda_{H1}$ and $\lambda_{H2}$ are the $L_{2}$ regularization rates for $\theta_{L}$ , $\theta_{H1}$ , and $\theta_{H2}$ .

### 2.5 Dataset generation, surrogate model training, and validation

We generated spatiotemporal datasets from FE simulations using the Latin hypercube sampling technique (LHS) to select sampling points from the parameter space Ξ (Table 1). Sampling points were drawn independently for the LF and HF datasets. We ran $N_{H}=25$ HF simulations and $N_{L}=300$ LF simulations. Thus, for each simulated quantity q ( $u_{x}$ , $u_{y}$ , $u_{z}$ , $p_{\mathrm{alv}}$ ) we obtained the corresponding output matrix $Z^{F,q}\in R^{N_{F}\times M}$ , from which we recall that M=K⋅T . For the HF datasets, K=4566 and K=5796 in the left and right lungs, respectively. For the LF datasets, K=318 and K=403 in the left and right lungs, respectively. For each output matrix, we performed SVD in order to obtain the truncated score matrices $Y_{k}^{F,q}\in R^{N_{F}\times k}$ that represent the reduced-order dataset of quantity q . We consider the first k principal components that capture at least 99% of the cumulative variance.

In constructing the rMFNN surrogate model, we tuned the following hyperparameters: regularization rate (λ) , number of hidden layers $\left(N_{\mathrm{layers}}\right)$ , and number of neurons per layer $\left(N_{\mathrm{neurons}}\right)$ . Based on preliminary experiments, we observed that relatively compact architectures already achieved satisfactory performance. To balance model expressiveness and computational feasibility, we fixed the LF network $NN_{L}$ with the following values { $\lambda_{L}:1e-3$ , $N_{\mathrm{neurons}}:60$ , $N_{\mathrm{layers}}:5$ }, and for both HF networks $NN_{H1}$ and $NN_{H2}$ we conducted a simultaneous grid-search for the values { $\lambda_{H1,H2}:\left[1e-1,1e-3\right]$ , $N_{\mathrm{neurons}}:\left[30,60\right]$ , $N_{\mathrm{layers}}:\left[3,6\right]$ }. To bound the cost of the grid-search, we constrained both HF networks to share the same regularization value, i.e., $\lambda_{H1}=\lambda_{H2}$ . For all three networks, we considered two activation functions: [Tanh, ReLU]. Therefore, we look for the architecture with the best performance among 16 possible combinations. We emphasize that we build separate surrogates for each quantity q ( $u_{x}$ , $u_{y}$ , $u_{z}$ , $p_{\mathrm{alv}}$ ), i.e., 4 for each lung (Figure 3). To implement the composite architecture we use the PyTorch library (Paszke et al., 2019). To minimize the loss function, we used the Adam optimizer (Kingma and Ba, 2014) with an initial learning rate α=1e−3 and 5,000 epochs, which was found to be sufficient to ensure convergence without signs of overfitting across the tested configurations.

To train and evaluate the rMFNN, we split the HF reduced dataset into 15 observations for training and 10 for testing. We used all 300 LF samples for training, resulting in a ratio of LF/HF training data of 20:1. We standardized all input values by subtracting the mean and dividing by their standard deviation. To assess the performance of the rMFNN, we compared it against a reference model trained solely on the reduced-order HF data, referred to as the single-fidelity neural network (rSFNN). We note that this model is equivalent to only keep $NN_{H2}$ in the composite architecture. For this rSFNN, we conducted the same grid-search configuration as the rMFNN counterpart. For model training and evaluation, we performed 3-fold cross-validation on the training HF data, using a train/validation split of 10/5 samples per fold. We trained, evaluated, and chose the model with the best performance in terms of the $R^{2}$ score. Since separate networks were trained for each output field ( $u_{x}$ , $u_{y}$ , $u_{z}$ , $p_{\mathrm{alv}}$ ), performance was evaluated using the $R^{2}$ score of the first principal component (PC1) of $u_{x}$ in the right lung, chosen as a representative output to enable consistent comparison between models

$$R^{2}=1-\frac{RSS_{PC1,u_{x}}}{TSS_{PC1,u_{x}}},$$ (22)

with

$$RSS_{PC1,u_{x}}=\frac{1}{N}\overset{N}{\underset{i=1}{\sum }}{\left(y-\hat{y}\right)}^{2},$$ (23)

$$TSS_{PC1,u_{x}}=\frac{1}{N}\overset{N}{\underset{i=1}{\sum }}{\left(y-\bar{y}\right)}^{2}.$$ (24)

In Equations 22–24, y are the HF ground truth PC1 values of the $u_{x}$ , $\bar{y}$ is the mean, $\hat{y}$ are the predicted PC1 values of the $u_{x}$ (by either the rSFNN or rMFNN model), and N is the number of evaluated samples. We note that the best possible $R^{2}$ score is 1.0 and it may take negative values since the model can be arbitrarily worse. We chose the evaluation on PC1 since it is the most relevant in terms of the explained variance. We remark that the best model architecture for $u_{x}$ is also used for the other networks ( $u_{y}$ , $u_{z}$ , $p_{\mathrm{alv}}$ ).

Then, we assessed the predictions of the optimal rSFNN and rMFNN architectures for ( $u_{x}$ , $u_{y}$ , $u_{z}$ , $p_{\mathrm{alv}}$ ) with respect to their corresponding HF testing data on both lungs. In addition to the $R^{2}$ score, we reported the mean absolute error (MAE) , defined as

$$\text{MAE}=\frac{1}{N}\overset{N}{\underset{i=1}{\sum }}|y-\hat{y}|,$$ (25)

In addition to Equation 25, we evaluated the reconstructed spatial response at peak of inspiration instant for both surrogates by means of the relative error (in percentage), defined in Equation 26.

$$\epsilon =|\frac{z_{t_{PIP}}-{\hat{z}}_{t_{PIP}}}{z_{t_{PIP}}}|\cdot 100\%,$$ (26)

To compare the effect of the HF dataset size on the performance of both rSFNN and rMFNN models, we introduce the equivalent high-fidelity training cost, denoted as $C_{eq}$ in Equation 27. This metric expresses the combined computational expense of using both HF and LF data (in rMFNN models) in terms of an equivalent number of HF samples. We define it as

$$C_{eq}=C_{H}+C_{L},$$ (27)

where $C_{H}=N_{H}$ is the number of HF samples used for training, and $C_{L}$ represents the additional cost contribution from obtaining LF samples, expressed in Equation 28 as the HF equivalent

$$C_{L}=\frac{N_{L}T_{L}}{T_{H}},$$ (28)

with $N_{L}$ denoting the number of LF samples and $T_{L}$ and $T_{H}$ the average computational time required by one LF and HF simulation, respectively. For rSFNN models, we note that $C_{L}=0$ since only HF samples are used for training.

All nonlinear finite element simulations and neural networks training were performed using a single Intel Core i7 processor with 16 Gb RAM.

## 3 Results

### 3.1 Numerical simulations of high- and low-fidelity lung poromechanical models

**FIGURE 4:**
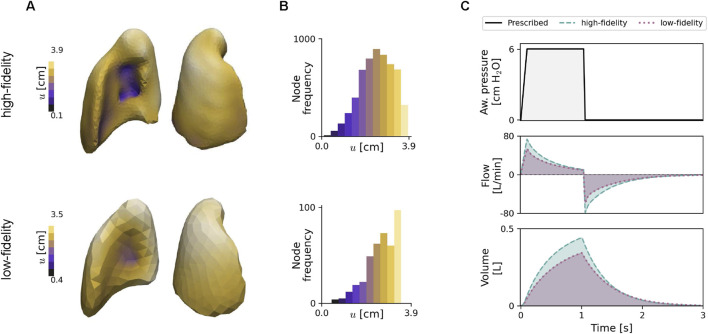
Numerical simulation of lung finite element models using the baseline values of model parameters. **(A)** Displacement field of the right lung at peak volume/end of inspiration time during PCV mode. **(B)** Frequency of the mesh nodes by their displacement value. **(C)** Computed signals over two respiratory cycles during the PCV mode: the physiological signals describe the time evolution of the prescribed airway pressure, airflow, and lung volume, which are shown for the HF (dashed) and LF models (dotted). The top and bottom rows in **(A,B)** correspond to HF and LF models, respectively.

Figure 4A shows the displacement magnitude field at the end of inspiration for HF and LF FE models using baseline parameter values. The lowest displacement values are located around the entrance of the airway. The frequency distributions for both models are shown in Figure 4B, where differences are readily observed: the LF distribution is more skewed to the left than the HF distribution. The airway pressure, airway flow, and lung volume signals postprocessed from simulations are reported in Figure 4C. The LF model resulted in lower amplitudes for volume and flow rate compared to the HF model response. In terms of computational cost, HF simulations took $T_{H}∼$ 2.7 h, whereas LF simulations typically required $T_{L}∼$ 2.9 min, resulting in a 55× speedup.

### 3.2 Singular value decomposition of spatiotemporal datasets

**FIGURE 5:**
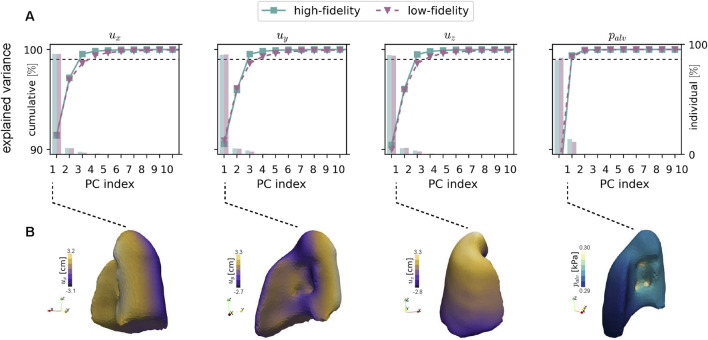
Analysis of variance from principal component analysis of HF and LF models. **(A)** For each quantity subplot ( $u_{x}$ , $u_{y}$ , $u_{z}$ , $p_{\mathrm{alv}}$ ), the left y-axis indicates the cumulative explained variance (line with markers), while the right y-axis shows the explained variance per individual component (bars). The 99% cumulative threshold is marked with a dashed line for reference. **(B)** Reconstructed spatial fields $u_{x}$ , $u_{y}$ , $u_{z}$ , and $p_{\mathrm{alv}}$ using the first principal component alone.

SVD resulted in principal components whose accumulated explained variance for each spatiotemporal dataset for fields ( $u_{x}$ , $u_{y}$ , $u_{z}$ , and $p_{\mathrm{alv}}$ ) are reported in Figure 5A for both fidelity levels and for the right lung. For left lung analysis, see Supplementary Figure S1. For the displacement field components of the HF and LF datasets, 99% of the cumulative explained variance (dashed line threshold) was reached when considering the first three principal components. For the case of the alveolar pressure field, only two principal components were required to achieve the same level of cumulative explained variance. Incremental variance associated to each principal component, displayed as colored bars in Figure 5A, shows that the first principal component (PC1) roughly explains 90% of the variance in the response of all fields. This trend is shared by both the left and right lung models. In addition, an asymptotic behavior after the fifth principal component $Y_{5}^{F}$ is observed in all cases. Figure 5B shows the spatial displacement and alveolar pressure fields that result from reconstructing them based on PC1 alone.

### 3.3 Hyperparameter tuning and performance assessment of surrogate models

Table 2 reports the results for the hyperparameter tuning step for both trained rSFNN and rMFNN models. The optimal rMFNN model has twice as many neurons and hidden layers as its rSFNN counterpart, and a lower regularization value λ . We note that this step results in the same value for the activation function for both models. The $R^{2}$ score was determined from a 3-fold cross-validation procedure using the training dataset and evaluated on the right lung first principal component (PC1) of $u_{x}$ , as described above. The rMFNN model resulted in a higher mean $R^{2}$ than the rSFNN model ( 90.6% vs. 71.6% ). Dispersion of the $R^{2}$ score was smaller for the rMFNN model than for the rSFNN model. The training time considers the entire cross-validation process for each network. The rMFNN model takes roughly 3 times longer to train than the rSFNN counterpart. The inference time considers both the prediction time of each neural network and the reconstruction time of the response to have the same format as the simulation outputs. Both models present similar inference times.

**TABLE 2:** Hyperparameter tuning of single-fidelity (rSFNN) and multi-fidelity (rMFNN) neural networks, training and inference times, and $R^{2}$ scores (mean and std. deviation) after the 3-fold cross-validation procedure on the right lung first principal component (PC1) predictions of $u_{x}$ .

	rSFNN	rMFNN
λ	1e-1	1e-3
$N_{\mathrm{neurons}}$	30	60
$N_{\mathrm{layers}}$	3	6
Activation	ReLU	ReLU
Training time [s]	547.2	1648.9
Inference time^a^ [s]	17.3	21.1
	$R^{2}$ [%]	$R^{2}$ [%]
Validation $\left(u_{x}\right)$	71.6 ± 16.9	90.6 ± 9.2

^a^ The inference time considers both the prediction time of the neural network and the reconstruction time of the response to have the same format as the simulation outputs.

Principal component predictions from the surrogate models were evaluated against HF ground truth on the test set using $R^{2}$ and MAE (Table 3). For rSFNN, $R^{2}$ scores were generally higher in the left lung, with the lowest performance observed in $p_{\mathrm{alv}}$ predictions (∼83%) . This pattern was mirrored in the MAE values across lungs. In contrast, the rMFNN model achieved $R^{2}$ scores above 93% across all cases and lungs, with consistently lower MAE than the rSFNN.

**TABLE 3:** Performance metrics of principal components predictions of the single-fidelity (rSFNN) and multi-fidelity (rMFNN) neural networks surrogate models.

		rSFNN		rMFNN	
		$R^{2}\left[\%\right]$	MAE *	$R^{2}\left[\%\right]$	MAE *
		Left – Right	Left – Right	Left – Right	Left – Right
Test	$u_{x}$	90.5–85.4	58.4–52.8	97.3–98.0	25.5–24.1
	$u_{y}$	87.6–81.9	46.4–88.8	97.2–93.7	23.9–28.4
	$u_{z}$	90.6–75.2	90.8–91.2	94.5–95.3	44.2–39.6
	$p_{\mathrm{alv}}$	83.4–88.8	21.9–19.7	99.6–99.7	3.3–3.9

^a^ For clarity and given that these are principal component predictions, we do not include the units in the MAE columns.

**FIGURE 6:**
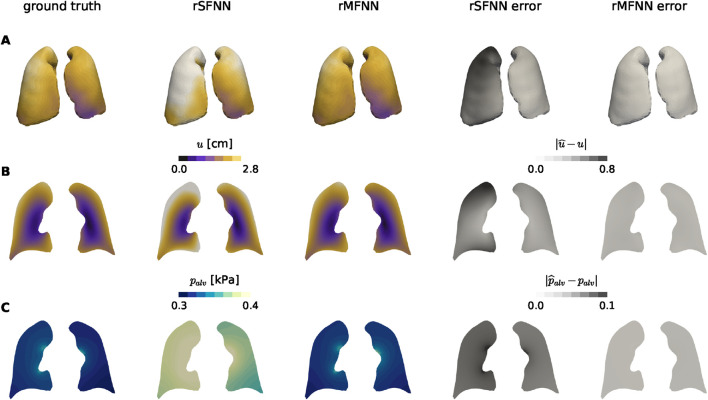
Performance analysis of displacement and alveolar pressure field predictions by the single-fidelity (rSFNN) and multi-fidelity (rMFNN) surrogate models. Ground truth fields are values obtained from a high-fidelity FE lung simulation. **(A)** Displacement magnitude fields on the surface of lung domains, **(B)** Displacement magnitude field on a coronal plane, and **(C)** Alveolar pressure field on a coronal plane. The last two rightmost columns show the absolute error between ground truth and surrogate model predictions.

**FIGURE 7:**
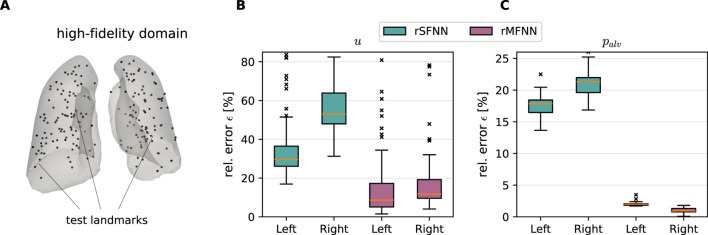
Nodal error assessment of predictions by the rSFNN and rMFNN surrogate models. **(A)** Landmark nodes inside the lung domains. Relative error distributions in the prediction of nodal values are shown for the displacement **(B)** and alveolar pressure fields **(C)** at landmark nodes.

Figure 6 shows the predicted displacement magnitude and alveolar pressure fields at peak inspiration instant for a case of the test set, using rSFNN and rMFNN models. Visually, rMFNN exhibits smaller absolute errors than rSFNN for both quantities, with predictions that are similar to the ground-truth fields. To quantify spatial prediction accuracy, we analyze pointwise errors at 100 randomly selected nodes (test landmarks) from the high-fidelity lung domain (Figure 7A). Relative error distributions for the left and right lungs are presented in Figure 7B, C for u and $p_{\mathrm{alv}}$ , respectively. Both subplots show consistently lower errors with the rMFNN model. A full error analysis across the test set is provided in Supplementary Figure S2.

### 3.4 Lung mechanics from surrogate models

**FIGURE 8:**
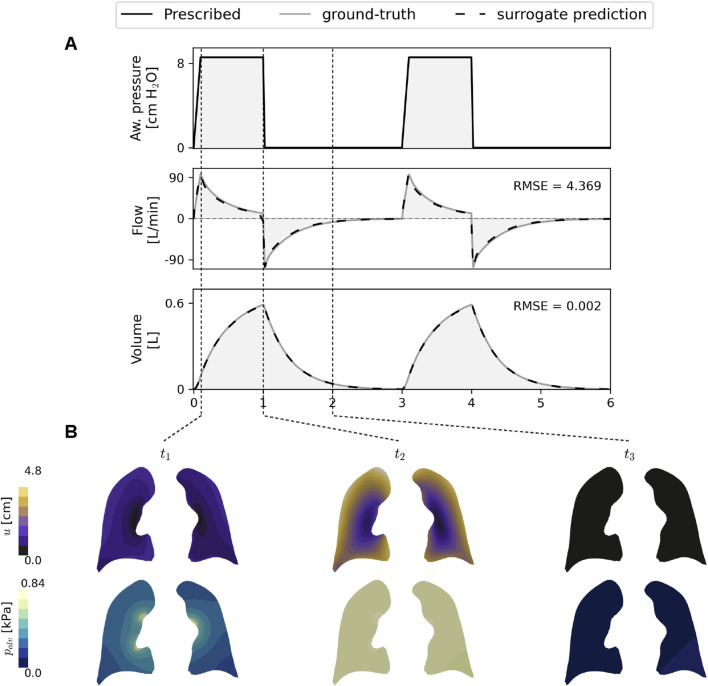
**(A)** Lung mechanics from multi-fidelity neural network (rMFNN) predictions. Dashed line denotes flow and volume from the rMFNN model. Solid lines show ground truth values from lung FE poromechanical simulations. **(B)** Displacement and alveolar pressure fields predicted by the rMFNN model along the respiratory cycle.

Figure 8A presents the temporal evolution of airway flow and lung volume for a representative test case, computed from rMFNN predictions (dashed line) using Equations 5, 6, alongside ground truth from FE simulations (solid line). The surrogate closely matches the ground truth, with RMSE values of 4.369L/min for flow and 0.002L for volume. RMSE results across the full test set are provided in Supplementary Table S1. Figure 8B shows displacement and alveolar pressure fields on coronal slices at peak inspiratory flow, peak expiratory flow, and mid-expiration.

### 3.5 Computational cost and effect of the dataset size in model performance

**FIGURE 9:**
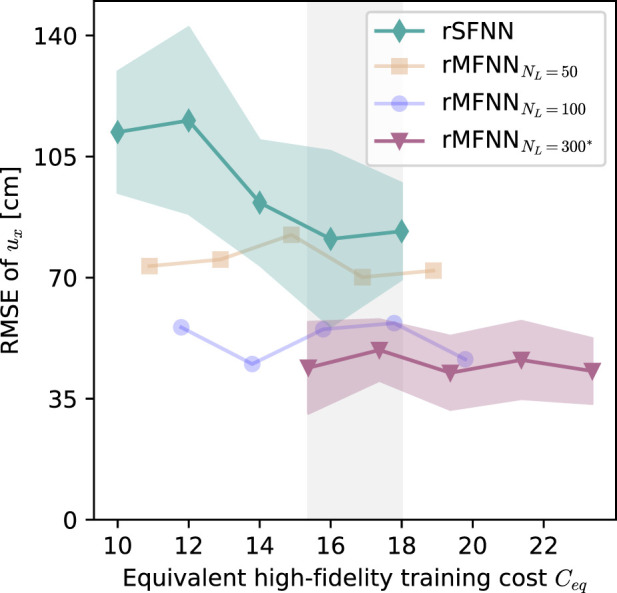
Comparison of model errors against the equivalent high-fidelity training computational cost $C_{eq}$ . Solid lines represent the mean RMSE of the predicted first principal components of $u_{x}$ for rSFNN and rMFNN models, trained with an increasing number of HF samples (from 10 to 15). Shaded regions indicate the standard deviation. The vertical gray box is the region where both models are comparable in terms of the training cost. The effect of low-fidelity dataset size on the rMFNN performance is shown for $N_{L}=50$ , $N_{L}=100$ , and $N_{L}=300$ training samples; for the first two, only the mean RMSE is shown for clarity. * corresponds to the multi-fidelity model used throughout this work.

Figure 9 shows the RMSE of the principal components predictions of $u_{x}$ versus computational training cost for rSFNN and rMFNN models. We visualize the errors of models trained with five different HF training sizes: from a reduced set of 10 up to the original 15 samples used in the surrogates presented throughout this work. The rMFNN cost accounts for the combined computational time of HF and LF data in terms of the equivalent high-fidelity training cost $\left(C_{eq}\right)$ . Given that each HF simulation takes $T_{H}∼162$ minutes and each LF simulation $T_{L}∼2.9$ minutes, the additional cost contribution from the obtained 300 LF samples are equivalent to $C_{L}=300\times 2.9/162=5.37$ HF simulations. This consideration results in a positive (right) shift in the equivalent HF training cost axis for the rMFNN case. Across all five cases, rMFNN consistently outperformed rSFNN. With 10 HF samples (i.e., equivalent HF training cost $C_{eq}=15.37$ samples), the rMFNN achieved a ∼61% reduction in the RMSE when compared to the rSFNN trained with the same number of samples ( 43.9±13.2 [cm] vs. 112.1±17.5 [cm]). An analysis of the training cost interval where both model intersect (shaded area between 15.37–18), we note that the rMFNN model always achieves a lower RMSE compared with the rSFNN model. We further note that the rMFNN model does not significantly decrease its RMSE as the HF training dataset size increases. In addition, we explore the effect of the LF dataset size on rMFNN performance, comparing models trained with 50 $\left(C_{L}=0.89\right)$ , 100 $\left(C_{L}=1.79\right)$ , and the original 300 LF samples. To improve readability, for the 50 and 100 LF cases we only report the mean RMSE. In general, the rMFNN RMSE decreased as the LF dataset size increased.

## 4 Discussion

In this work, we leverage multi-fidelity deep learning and dimensionality reduction to construct efficient and accurate surrogate models of the spatiotemporal poromechanical response of lungs connected to mechanical ventilation. A crucial component of our framework is the dimensionality reduction of spatiotemporal datasets used to train NN models. We found that as few as 5 principal components are sufficient to capture over 99% of the cumulative variance in both the displacement and alveolar pressure fields (Figure 5). This reduced set of principal components translates into a convenient complexity reduction that has also been reported in the literature when modeling other physical systems. Indeed, the optimal number of principal components ranged between 3 and 11 in the prediction of stress fields in patient-specific skull geometries (Lee et al., 2018), in the numerical simulation of fluid velocity fields in wake models (Pawar et al., 2022b), in the prediction of soft tissue deformation in childbirth simulation (Nguyen-Le et al., 2023), and in the construction of temperature and pressure fields in thermomechanical simulations of clutches (Schneider et al., 2024). We remark that these contributions focus on reducing the dimensionality of the spatial domain only, which highlights the novelty of our work in incorporating the temporal dimension into datasets that are analyzed using SVD.

A key objective of our work is the construction of a reduced multi-fidelity surrogate model, which we have shown delivers more accurate spatial predictions of the displacement and alveolar pressure fields than the single-fidelity model using the same number of HF samples for training (Figure 6). The rMFNN model consistently achieved test $R^{2}$ scores above 93% and in average 97% for all cases ( $u_{x}$ , $u_{y}$ , $u_{z}$ , and $p_{\mathrm{alv}}$ ), outperforming the rSFNN model 3. Further, the rMFNN results in a MAE reduction of 56% and 83% in the prediction of displacements and alveolar pressure principal components. Further, the rMFNN achieved an average reduction of the median relative error on the test set of roughly 15% and 8% in the displacement and alveolar pressure fields, respectively (Figure 7; Supplementary Figure S2). However, in some cases, there are outliers with high errors in the nodal predictions, which we argue is due to the inevitable loss of SVD information, as well as the fact that the HF dataset is very small. These results highlight the benefit of considering a multi-fidelity architecture that benefits from adding LF data to the training step of the surrogate model. This trend is shared by other contributions in the literature. Gaussian Process surrogate models trained to predict spatial growth during tissue expansion have achieved relative errors below 1% between the single- and multi-fidelity models (Lee et al., 2020), on domains discretized with at most 100 regions. Multi-fidelity convolutional neural networks have been proposed to predict temperature fields governed by a linear heat equation in 2D square domains, resulting in a 62% reduction in MAE with only 10 HF data samples (Zhang et al., 2023). A long short-term memory architecture to predict fluid wake physics on rectangular 2D domains results in relative error reductions of up to 15% compared to single-fidelity models when predicting velocity and pressure fields (Conti et al., 2024). For a similar physical problem, surrogate models that consider PCA and multi-fidelity NN have demonstrated high prediction capabilities (Pawar et al., 2022b). Relative errors below 1% have been reported when compared to direct numerical simulations of the 2D domain of a flow past a cylinder. Further, the lung mechanics response to MV computed from rMFNN predictions shows a very high agreement with HF simulations (Figure 8A), with RMSE values that are similar to those offered by ML models specifically trained to predict lung mechanics (Barahona et al., 2024). Related to our combined deep learning and dimensionality reduction approach is the PCA-Net (Bhattacharya et al., 2021), an operator learning technique that uses PCA to reduce the dimensionality of both input and output spaces and then uses neural networks to approximate a map between the resulting finite-dimensional latent spaces. However, their implementation is in a single-fidelity setting. We conclude that our rMFNN model compares well to previously reported contributions while proving advantageous in that unstructured 3D domains can be considered for surrogate modeling of highly non-linear spatiotemporal problems, all crucial features that enable personalized predictions that have not been addressed in the past.

One key concern about DL models is the training computing effort. Our rMFNN model naturally results in a higher training computational cost than the rSFNN model (Table 2), an aspect frequently reported in the development of multi-fidelity surrogate models (Lee et al., 2020; Zhang et al., 2023; Barahona et al., 2024). This higher cost is attributed to the additional effort in creating LF samples for the training dataset and the larger number of parameters involved in composite NN architectures (Figure 3). One of the key results of this work is that this additional computational cost is well rewarded in terms of higher accuracy of surrogate prediction. Indeed, for the same equivalent training cost, the rMFNN model results in a considerably lower RMSE than the rSFNN model, offering roughly a 50% reduction in error for the same training cost (Figure 9. We also remark that the LF training dataset size has a marked effect on reducing the prediction error. This trend is clearly observed when increasing the LF sample size from 50 to 100. However, further increasing the LF sample size above 100 may not efficiently decrease the prediction error. This observation suggests that the LF dataset has an optimal size, which is likely to be problem-dependent and deserves a case-by-case analysis.

When analyzing the computational cost of predicting the spatiotemporal poromechanical lung response, our rMFNN model offers inference times that roughly take 21 s, which represents a speed-up of 462 × when compared to HF (direct numerical) FE simulations. While we did not find other works on lung poromechanics, acceleration through DL surrogate modeling has been applied to other physiological systems of the human body. Speed-ups of 41 × have been achieved in predicting the spatiotemporal electrophysiological behavior of the left ventricle (Fresca et al., 2020), which shares similar problem complexity and dimensionality as our lung poromechanical model. Siamese neural networks of 3D breast models have achieved speed-ups of 82.5 × in the prediction of the displacement fields when compared to FE models (Dang Vu et al., 2021). Random-forest regression combined with PCA dimensionality reduction delivers speed-ups of 18 × when predicting the 3D displacement fields in human liver geometries (Lorente et al., 2017). Higher speed-ups are obtained in static problems using shell models for predicting the mechanics of large vessels, where speed-ups up to 1800 × have been reported in surrogate models built using PCA and NNs (Liang et al., 2018). Based on these examples, we conclude that our rMFNN lung model offers an attractive speed-up for fully 3D non-linear time-dependent problems. Further, achieving full lung spatiotemporal predictions in less than a minute offers a computational tool that holds promise in meeting the time requirements of clinical applications.

Our results demonstrate that combining dimensionality reduction with deep learning can be an effective strategy for approximating spatiotemporal lung simulations. There are several opportunities for improvement that can further increase the potential of our rMFNN lung model. First, the dimensionality reduction may benefit from exploring recent techniques such as autoencoders, which find interesting applications in biomechanical modeling (Fresca et al., 2020; Conti et al., 2024; Deshpande et al., 2025). Since an autoencoder is a neural network, it can provide greater flexibility and be easily adapted to the DL pipeline. Although autoencoders have shown an accuracy similar to that achieved by PCA and SVD (Bourlard and Kabil, 2022; Cacciarelli and Kulahci, 2023), their efficiency in speed-up with respect to the aforementioned techniques is still a relatively unexplored avenue of research (Fournier and Aloise, 2019). Second, while using only 15 HF samples for training may lead to overfitting or underfitting, the multifidelity framework is designed precisely to mitigate this limitation by leveraging the abundance of LF data to guide learning. Increasing the number of HF samples would reduce the risk of overfitting, but would also diminish the computational advantage and the purpose behind the multifidelity approach. However, we acknowledge that a sufficient number of HF test samples is required to properly validate the model and ensure its generalization capability. Future efforts should aim to optimize the sampling strategy, particularly to minimize unnecessary evaluations of the computationally expensive high-fidelity model Lee et al. (2020); Gander et al. (2022). Third, our rMFNN framework operates with fixed lung geometries, necessitating retraining when a different lung anatomy is analyzed. This limitation can be addressed by considering DL architectures that embed the topology of the physical system, such as Graph Neural Networks (GNNs) (Scarselli et al., 2008). Current applications of GNNs to biological systems include modeling cardiac mechanics (Dalton et al., 2022), brain shift simulations (Salehi and Giannacopoulos, 2022), cartilage and soft tissue mechanics (Sajjadinia et al., 2022), and foot biomechanical simulations (Kang et al., 2025). We foresee that an extension to lung poromechanics can leverage the geometrical flexibility provided by GNN modeling. Alternatively, operator learning techniques such as neural operators (NOs) have shown potential to learn and emulate PDEs while being discretization-invariant, which could be explored with a multi-fidelity setting Azizzadenesheli et al. (2024). Fourth, we note that the rMFNN model needs to include more variables to better represent clinical conditions. In particular, respiratory rate, tidal volume, and positive end-expiratory pressure are all important variables in MV that change from patient to patient. Further, lungs can display mechanical heterogeneity, particularly in pathological cases, which is not represented by a single set of constitutive parameters. Gravity is another important parameter known to have effects on both regional and global lung response Bettinelli et al. (2002); Hurtado et al. (2017). Therefore, future contributions should increase the number of variables to adequately capture clinical scenarios and pulmonary conditions such as respiratory distress and pulmonary emphysema (Hurtado et al., 2020; Villa et al., 2024; Nelson et al., 2024). Lastly, we remark that the poromechanical framework considered in the generation of spatiotemporal datasets only considers the non-linear hyperelastic behavior of lung tissue through phenomenological constitutive models. This approach, while practical and effective, cannot directly account for alveolar structural features (Concha et al., 2018) nor for the hysteretic response of alveolar tissue (Avilés-Rojas and Hurtado, 2025). Future contributions will benefit from incorporating multiscale tissue models that address the inelastic response of alveolar tissue. These and other improvements will contribute to the construction of predictive surrogate models that can greatly impact clinical applications in respiratory medicine.

## Data Availability

The raw data supporting the conclusions of this article are available from the corresponding author upon reasonable request.
