# Supplementary material for: Multifidelity deep learning modeling of spatiotemporal lung mechanics
Source: Front Physiol. 2025 Sep 24;16:1661418. doi: 10.3389/fphys.2025.1661418 (PMC12504473; doi:10.3389/fphys.2025.1661418)
Supplement: Supplementary file 1 [file DataSheet1.pdf]

Supplementary Material

1 SUPPLEMENTARY TABLES AND FIGURES

1.1 Figures

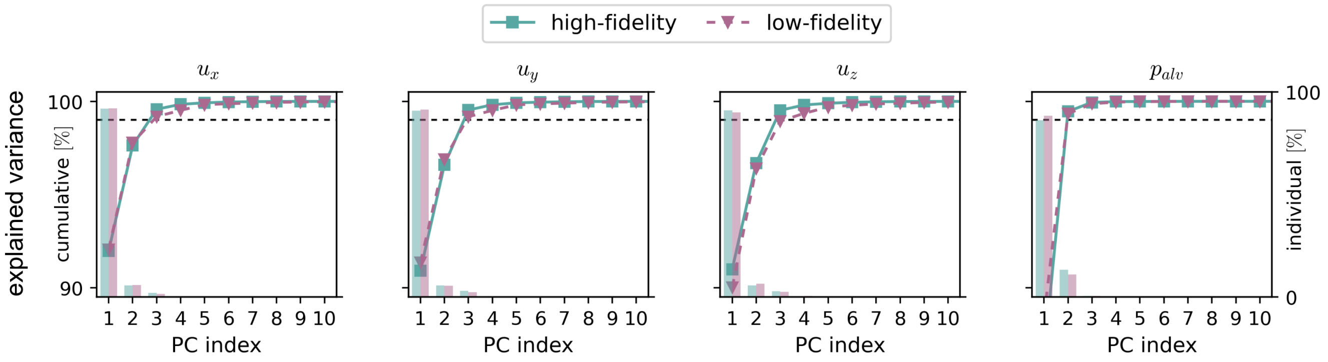

Figure S1. Analysis of variance from principal component analysis of HF and LF models. (a) Cumulative explained variance vs. number of principal components for displacement and pressure fields in the left lung. Bars show the incremental variance associated with a single principal component. The first three columns correspond to displacement component fields u_x, u_y, u_z, while the last column reports the case of alveolar pressure field p_{alv}. The 99% cumulative threshold is marked with a dashed line for reference. (b) spatial fields u_x, u_y, u_z, and p_alv reconstructed using the first principal component alone.

1.2 Tables

		**MFNN RMSE**	
Test case	$\bar{P}_{pip}$ cm H_20	Flow [L/min]	Volume [L]
1	4.768	1.457	0.001
2	4.508	2.891	0.001
3	8.417	4.369	0.002
4	3.918	1.803	0.001
5	8.009	3.609	0.001
6	3.976	2.440	0.002
7	7.785	3.329	0.004
8	3.259	2.295	0.001
9	5.328	3.170	0.003
10	3.565	2.397	0.001
	Mean	0.001	0.002

Table S1. RMSE results for Flow and Volume signals across all the cases of the test set.

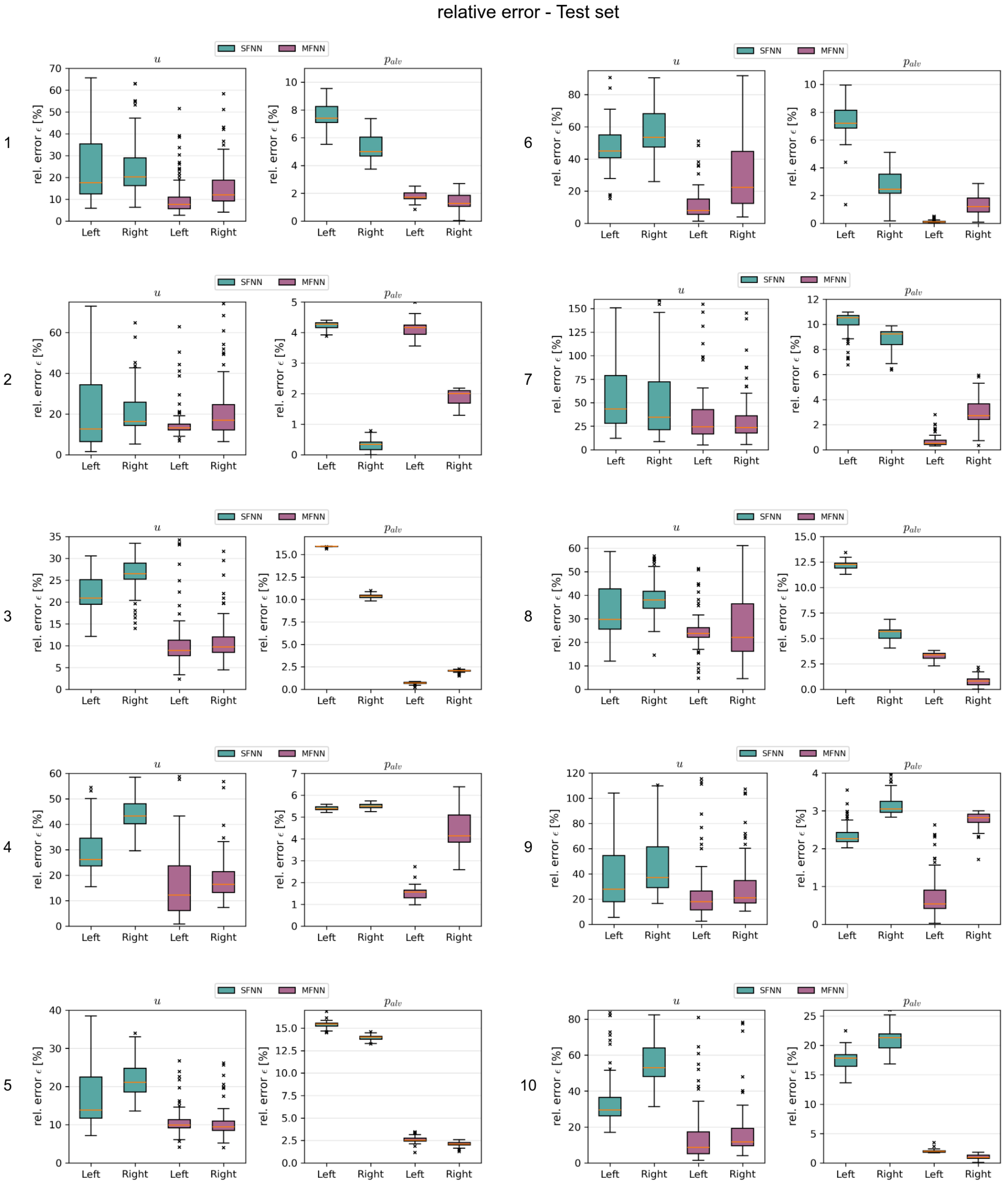

Figure S2. Boxplots for the absolute error in the testing set of the SFNN and MFNN predictions with respect to HF values in the testing markers. We show the error in the displacement field and the alveolar pressure during peak volume instant for both left and right lungs. Horizontal yellow lines denote the median of the error in each box.
